# Sex-dependent dysregulation of the gut-brain NPYergic system in a mouse model of autism spectrum disorder

**DOI:** 10.1038/s41598-026-42601-0

**Published:** 2026-03-04

**Authors:** Beatriz Martins, João Martins, Miguel Castelo-Branco, Joana Gonçalves

**Affiliations:** 1 Coimbra Institute for Biomedical Imaging and Translational Research (CIBIT), University of Coimbra, Coimbra, Portugal; 2 Institute of Nuclear Sciences Applied to Health (ICNAS), University of Coimbra, Coimbra, Portugal; 3 Institute of Physiology, Faculty of Medicine, University of Coimbra, Coimbra, Portugal; 4 Faculty of Pharmacy, University of Coimbra, Coimbra, Portugal

**Keywords:** Gut-brain, Neuropeptide Y, Sex dimorphism, Autism spectrum disorder, Autism spectrum disorders, Molecular neuroscience, Animal disease models, Reverse transcription polymerase chain reaction

## Abstract

The microbiome-gut-brain axis has been increasingly recognized for its role in the pathophysiology of autism spectrum disorder (ASD), yet the underlying molecular mechanisms remain poorly understood. Neuropeptide Y (NPY), a key modulator of gut-brain communication, may play a pivotal role in this axis. This study investigated the sex-specific molecular profile of the NPY system in gut-brain communication via a genetic mouse model of ASD, the *Nf1*^+*/-*^ mice. Quantitative real-time PCR was performed to assess the expression of NPY and its receptor transcripts in the amygdala, hippocampus, prefrontal cortex and intestinal tissue of juvenile male and female *Nf1*^+*/-*^ mice. Additionally, gut microbiota analysis focused on *Lactobacillus* species in stool samples. Special emphasis was placed on sex differences, an area underexplored in ASD research. Sex-specific differences in NPY and its receptor expression were observed in both the brain and intestinal tissues of *Nf1*^+*/-*^ mice. In mutant females, estrous cycle fluctuations were partly associated with changes in the NPY system. Notably, distinct correlations between the brain and intestinal NPY systems were identified in both sexes of wild-type (WT) and *Nf1*^+*/-*^ mice. Microbiota analysis revealed sex-dependent alterations in *Lactobacillus* abundance, which correlated with the intestinal NPY system. Importantly, the Y2 receptor exhibited sex-specific expression patterns in both the gut and brain of *Nf1*^+*/-*^ mice. This study provides novel evidence that the NPY system may play a critical role in gut-brain communication in ASD, with sex-dependent alterations in both the brain and gut. The intestinal Y2 receptor has emerged as a potential molecular biomarker for ASD, underscoring the importance of incorporating sex as a biological variable in future ASD research.

**Supplementary Information:**

The online version contains supplementary material available at 10.1038/s41598-026-42601-0.

## Introduction

According to the World Health Organization, one in every 100 children is affected by autism spectrum disorder (ASD)^1^. The core symptoms of ASD are characterized by abnormal social functioning and repetitive and restricted patterns of behavior. However, clinical data have shown that boys have more externalizing disorders such as hyperactivity, aggression, and impulsivity^2–5^. In contrast, girls with ASD present associated problems such as depression, anxiety, sleeping, or other emotional symptoms^2,4^. In addition to this heterogeneity, genetic factors play a central role in ASD etiology. Mutations in the *Nf1* gene, which cause Neurofibromatosis Type 1 (NF1), have been associated with a higher prevalence of ASD traits, including social and cognitive difficulties^6–8^. The *Nf1*^+*/–*^ mouse, although not a canonical ASD model, reproduces several of these phenotypes^9,10^ and shows underlying altered neurotransmission^11^ that parallels mechanisms implicated in ASD pathophysiology. For this reason, the *Nf1*^+*/–*^ model provides a valuable tool to investigate ASD-related neurodevelopmental alterations.

Gastrointestinal (GI) disorders co-occur in 83–91% of children with ASD and are increasingly recognized as factors influencing symptom severity^12–14^. Dysbiosis and impaired intestinal barrier functions have been documented in multiple preclinical models with autism-associated gene mutations, which are correlated with abnormalities in stereotypic behaviors, communication, sensorimotor function, and anxiety^15,16^. Importantly, interventions such as probiotic treatments have been shown to restore GI function and alleviate some behavioral impairments associated with ASD^17,18^. These findings underscore the critical role of the gut microbiome in modulating neural circuits and behavior.

The gut microbiota facilitates complex bidirectional communication between the gut and the brain, which is mediated in part by neuropeptides and neurotransmitters^19,20^. Several neuropeptides, such as neuropeptide Y (NPY), are secreted from enteroendocrine cells in the gastrointestinal tract, allowing this biological process^21^. NPY, along with peptide YY (PYY), belongs to a family of endocrine peptides that act primarily through G protein-coupled receptors (Y1, Y2, Y4, and Y5) with region-specific effects^22^. Although NPY is expressed in the gut-brain axis, PYY is almost exclusively expressed by endocrine cells of the digestive system. Preclinical and clinical studies suggest that NPY exerts anxiolytic effects, particularly via Y2 receptor signaling^23–28^. While NPY is produced in both the central nervous system (CNS) and the enteric nervous system (ENS), its role in maintaining gut microbiota homeostasis remains underexplored.

Given the widespread expression of NPY and its receptors in the gut-brain axis, investigations of its role in this context and its potential implications for ASD pathogenesis are crucial. Furthermore, the role of NPY in ASD pathogenesis remains fully unexplored. Here, we hypothesize that alterations in the brain and gut NPY system expression are associated with gut dysbiosis in ASD. This study aims to elucidate the interplay between neuropeptides and the gut microbiome, advancing our understanding of their roles in ASD.

## Methods

### Animals

Female and male *Nf1*^+*/-*^ mice were obtained from mutant animals C57BL/6N mice backcrossed with 129T2/SvEmsJ. As a control, we used no-transgenic littermates.

Tails were cut at postnatal day (PND) 4–5, and genotypes were determined via PCR reaction (primers used were: NF1 P132-TTCAATACCTGCCCAAGG; P133-ATTGCCAATGACAA; P134-GGTATTGAATTGAAGCAC). A total of fifteen-seven animals were used in this study, distributed across the experimental groups as detailed in the figure legends. The animals remained undisturbed with progenitors until PND21. Offspring animals were housed (2–4 per cage) at PND21 on a 12 h light/dark cycle in ICNAS/University of Coimbra animal facilities. The animals were euthanized by cervical dislocation at PND 60. The hippocampus, amygdala, prefrontal cortex (PFC), intestine, and stool were collected in RNAlater® Solution (Invitrogen) for molecular and biochemical analysis. The samples were stored at −80ºC until further processing. The experiments were carried out following the European Union Council Directive (2010/63/EU), the National Regulations (8/2003), and the Internal Review Board of the University of Coimbra. All results are reported in consistency with ARRIVE (animal research: reporting in vivo experiments) guidelines.

### Brain and intestinal DNA extraction

After the animals were euthanized, the hippocampus, PFC and amygdala were isolated, and RNA was extracted with RNeasy Lipid Tissue Kit (Qiagen, Hilden, Germany), according to the manufacturer’s instructions. The purification included a DNase treatment using the RNase-free DNase Set (Qiagen, Hilden, Germany). Intestinal RNA was extracted using NZY Total RNA isolation kit (NZYtech, Portugal) and was followed by a DNAse digestion step to remove any contaminating genomic DNA.

### RNA quality assessment and cDNA synthesis

The DNA and RNA yields and purity of the extracts were measured photometrically using Nanodrop ND-1000 spectrophotometer (LabTech UK). To determine DNA and RNA purity, the A260 nm/A280 nm ratio of each sample was determined. For cDNA synthesis, 1 µg of RNA was amplified using iScript cDNA Reverse Transcriptase Kit (Bio-Rad Laboratories, Hercules, CA). The reactions were carried out in a thermocycler (Bio-Rad Laboratories, Hercules, CA) as follows: 25ºC for 5 min, 46º for 20 min and 95ºC for 1 min for 40 cycles. The samples were stored at −20ºC until further processing.

### Stool collection and microbial DNA extraction

Stool samples from the animals were collected at PND60, and DNA was extracted using RNeasy Power Microbiome kit (QIAGEN GmbH, Hilden, Germany) with minor adaptations. Briefly, an additional heating step of 95 °C for 10 min after step 4 was added because this step was previously shown to increase the DNA yield^29^. Genomic DNA was eluted in 100 µL of RNase-free H_2_O and quantified using Nanodrop. For downstream applications, samples were diluted to 50 ng/µL.

### Real-time qPCR analysis

Real-time quantitative polymerase chain reaction (RT-qPCR) of microbial DNA, and brain and intestinal cDNA was performed using primers listed in the Additional File, Table 1. RT-qPCR was performed in mixture containing SsoAdvanced Universal SYBR Green Supermix (Bio-rad Laboratories, Hercules, CA), 500 nM primers and 20 ng of cDNA or 100 ng of bacterial DNA. The amount of cDNA input for qPCR (20 ng) was calculated based on the initial RNA quantification, following the manufacturer’s recommendations for the reverse transcription kit. All reactions were multiplexed with the housekeeping genes *B2M, YWHAZ* and *HRPT* (for the brain and intestine) and 16S (for intestinal bacteria). Reactions were carried out with a CFX PCR System (Bio-Rad Laboratories, Hercules, CA). The expression of each gene was compared between groups using the 2^-ΔΔCT^ method^30^. For all the qPCR reactions, every sample was amplified in duplicate. To ensure specific amplification, qPCR mixtures without DNA were included as negative controls. Additionally, to validate the specificity and efficiency of the amplification of the primers and probe, qPCR amplification was previously performed via a tenfold dilution series from template DNA, and an efficiency ranging from 90 to 110% was achieved. The choice of housekeeping genes was first based on primer efficiency comparisons between NF1 and WT samples, followed by evaluation of gene stability using RefFinder^31^.

### Determination of the estrous cycle

A vaginal smear was collected before sacrificing the females, and cells were transferred to a dry glass slide and dried overnight ^32^. The glass slides were stained with crystal violet and estrous cycle stages were determined based on the presence or absence of specific cells according to Ajayi et al., 2020^33^). All the samples were examined under light microscopy, using a Zeiss Axioplan 2 microscope (Carl Zeiss Microscopy, LLC, NY, USA), and images were captured using a digital microscope camera (Leica DFC450, Leica Microsystems, Wetzlar, Germany) at 200 × magnification. Proestrus and estrus were considered the follicular phase and metestrus and diestrus were considered the luteal phase. The estimation of the phase of the estrous cycle is based on the proportions of leucocytes, cornified epithelial and nucleated epithelial cells in the vaginal secretions^33^. Representative images are shown in Additional files – Additional Fig. 1.

**Fig. 1:**
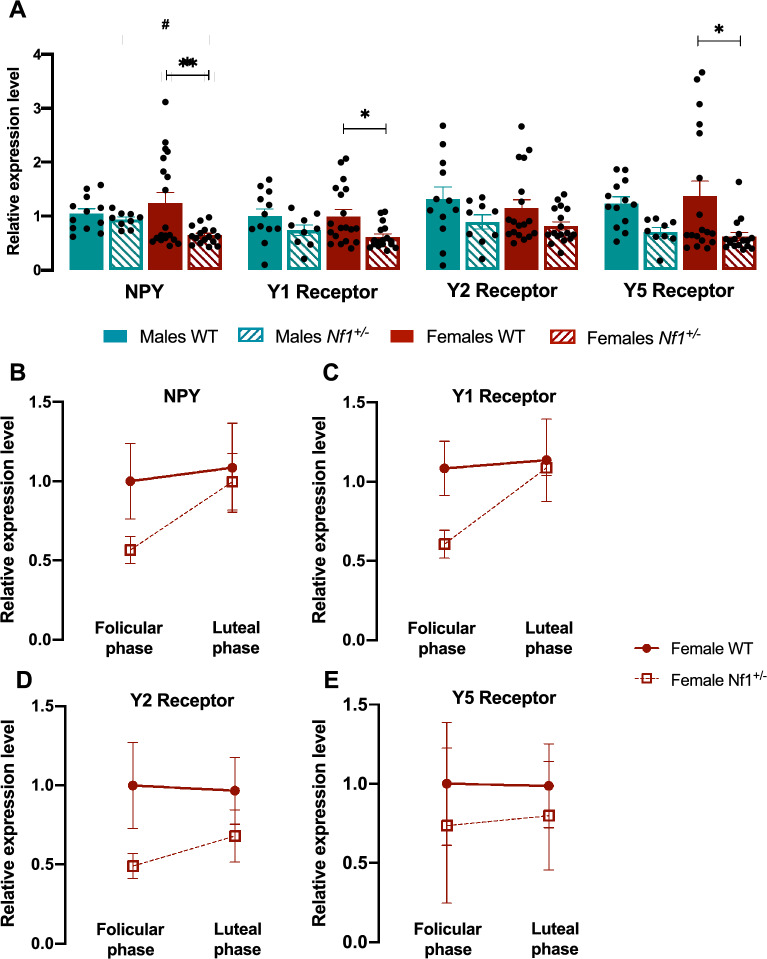
Cortical NPY system expression in male and female *Nf1*^+*/*-^ mouse. (**A**) Relative mRNA expression of *NPY* and their receptors *Y1*, *Y2* and *Y5* in prefrontal cortex (PFC) of WT and mutant males and females. While no alterations were observed in *Nf1*^+*/-*^ males, mutant females exhibited a significant decrease in NPY, Y1, and Y5 receptor levels. Graphs depict individual values, mean, and standard error (n = 9 – 19 per group). One-way ANOVA followed by Tukey’s post-hoc test was used for multiple comparisons. *p < 0.05, **p < 0.01. (**B**–**E**) mRNA expression of (**B**) NPY, (**C**) Y1, (**D**) Y2, and (**E**) Y5 across the estrous cycle in *Nf1*^+*/-*^ females. No significant differences were observed. Graphs depict mean, and standard error (n = 6 – 3 per group). One-way ANOVA followed by Tukey’s post-hoc test was used for multiple comparisons. *p < 0.05, **p < 0.01, ***p < 0.001.

### Statistical analysis

Data analysis was performed in GraphPad Prism version 9 (GraphPad Software, San Diego, CA, USA). The outliers were identified and excluded after ROUT analysis (Q = 5%), which was applied conservatively to minimize the influence of extreme values on group means. Normality tests were performed via the Shapiro–Wilk test. The data are expressed as the mean ± SEM. One-way ANOVA followed by Tukey’s post-hoc test was used for multiple comparisons. The correlations were performed using Spearman’s analysis of absolute values of ΔC_q_. The correlation between each indicator was compared, and the correlation heat map was drawn using ChiPlot (https://www.chiplot.online/) (accessed on 12 March 2025). Since this work was intended to be hypothesis-generating, no formal multiple comparisons correction was applied across genes, brain regions, or estrous phases. The exact n distribution across the experimental groups, the values of mean ± SEM, p values and 95% confidence intervals of means are presented in additional files—Tables 2, 3, 4, 5.

## Results

### The levels of the brain NPY system are sex dependent in Nf1^+/-^ mice

Several neuropsychiatric disorders, such as depression and schizophrenia^34^, have been linked to changes in NPY levels^35,36^. However, until now, possible alterations in the central NPY system under autistic conditions have remained unknown. Since early developmental dysfunction in the amygdala and hippocampus in individuals with ASD may cause a breakdown in brain connectivity normally recruited during complex cognitive tasks and trigger abnormal PFC development^37^, we focused our investigation on these three brain regions. The NPY system gene expression, namely the levels of *NPY* and its receptors *Y1*, *Y2* and *Y5,* was analyzed. We found both region- and sex-dependent changes in the expression of these genes. Cortical levels of *NPY*, as well as *Y1* and *Y5,* were lower in mutant females than in their WT littermates (Fig. 1A; *NPY* – Female WT: 1.242 ± 0.193, Female *Nf1*^+*/-*^: 0.645 ± 0.041, p = 0.0085; *Y1* – Female WT: 0.999 ± 0.036, Female *Nf1*^+*/-*^: 0.616 ± 0.054, p = 0.0358; *Y5* – Female WT: 1.372 ± 0.276, Female *Nf1*^+*/-*^: 0.626 ± 0.074, p = 0.0175). Interestingly, no sex differences in mRNA expression were detected between male and female *Nf1*^+*/-*^ mice (Fig. 1A). Additionally, no changes were detected in the cortical male NPY or *Y2* receptor levels (Fig. 1A). In vitro and in vivo studies suggest that NPY gene expression and its receptors are modulated by gonadotropic sex hormones^38,39^. Therefore, it is also important to consider the estrous cycle and fluctuating hormone levels when investigating the NPY system in females. However, no changes were detected in cortical NPY or its receptors between the follicular and luteal phases (Fig. 1B–E).

**Fig. 2:**
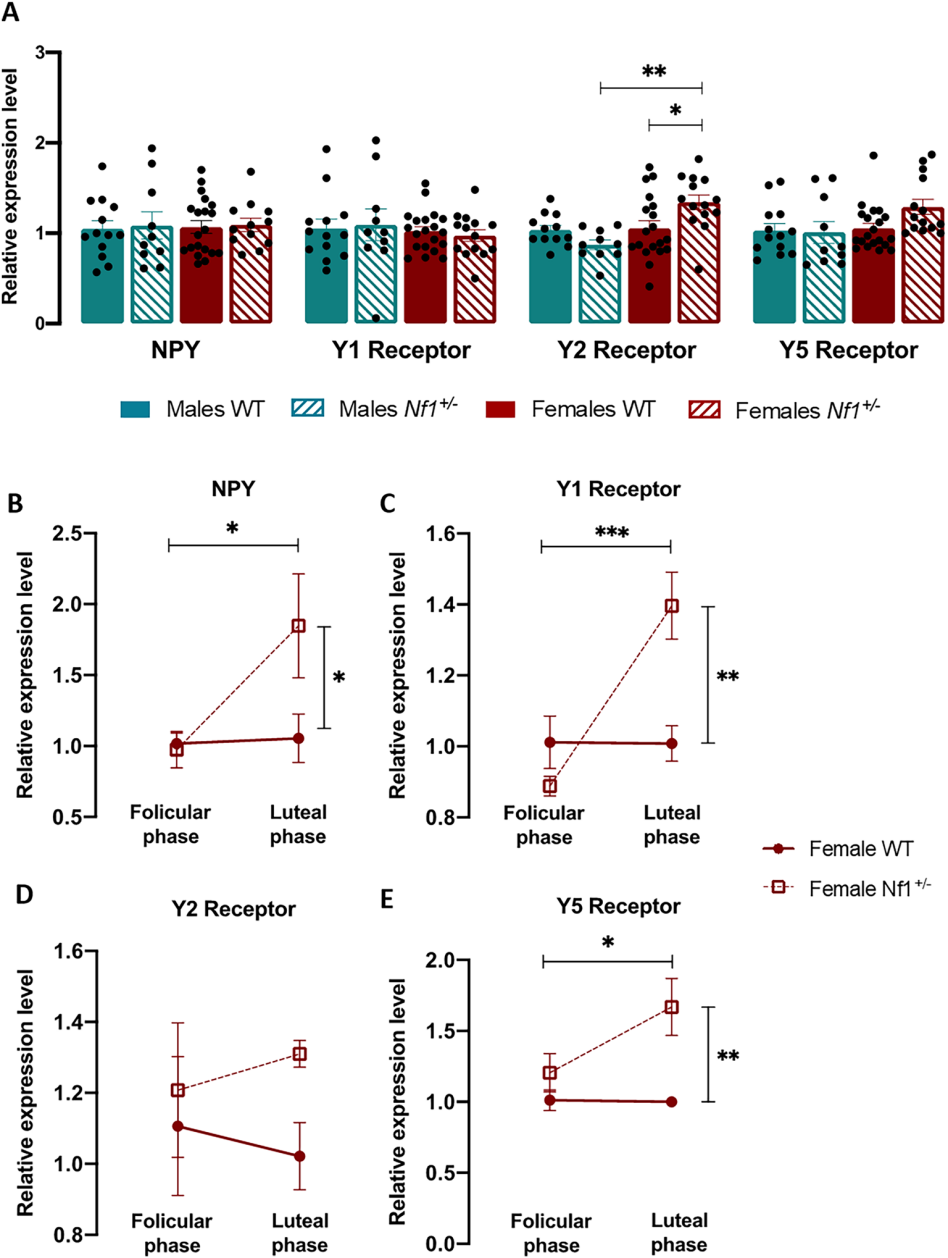
Expression of the NPY system in the amygdala of male and female *Nf1*^+*/-*^ mice. (**A**) Relative mRNA expression of NPY and its receptors (Y1, Y2, and Y5) in the amygdala of WT and *Nf1*^+*/-*^ males and females. A significant upregulation of Y2 receptor mRNA was observed only in *Nf1*^+*/-*^ females compared to both mutant males and WT littermates. Graphs depict individual values, mean, and standard error (n = 10 – 19 per group). *p < 0.05. (**B**–**E**) mRNA expression of (**B**) *NPY*, (**C**) *Y1*, (**D**) *Y2*, and (**E**) *Y5* across the estrous cycle in *Nf1*^+*/-*^ females. NPY, Y1, and Y5 showed upregulation during the luteal phase, while no changes were detected in *Y2* expression. Graphs depict mean, and standard error (n = 6—3). One-way ANOVA followed by Tukey’s post-hoc test was used for multiple comparisons. *p < 0.05, **p < 0.01, ***p < 0.001.

In the amygdala, an increase in *Y2* receptor levels was detected only in *Nf1*^+*/-*^ females compared with both transgenic males and WT littermates (Fig. 2A; *Y2* – Male *Nf1*^+*/-*^: 0.873 ± 0.054, Female *Nf1*^+*/-*^: 1.343 ± 0.080, p = 0.001; Female WT: 1.056 ± 0.085, p = 0.0277). No changes were observed in *NPY, Y1* or *Y5* expressions were observed between the experimental groups (Fig. 2A). Regarding the influence of the estrous cycle, we found that only the *Y2* receptor was not affected by hormonal fluctuations (Fig. 2D). Although, *NPY, Y1* and *Y5* levels did not change between follicular and luteal phases in WT females, their levels in females *Nf1*^+*/-*^ increased significantly during luteal phase (*NPY*—Female *Nf1*^+*/-*^ follicular phase: 0.974 ± 0.129, Female *Nf1*^+*/-*^ luteal phase: 1.847 ± 0.367, p = 0.037; Female WT luteal phase: 1.055 ± 0.172, p = 0.024, Fig. 2B; *Y1*—Female *Nf1*^+*/-*^ follicular phase: 0.888 ± 0.028, Female *Nf1*^+*/-*^ luteal phase: 1.397 ± 0.367, p = 0.0006; Female WT luteal phase: 1.012 ± 0.074, p = 0.0054, Fig. 2C; *Y5*—Female *Nf1*^+*/-*^ follicular phase: 1.206 ± 0.135, Female *Nf1*^+*/-*^ luteal phase: 1.670 ± 0.200, p = 0.044; Female WT luteal phase: 1.000 ± 0.023, p = 0.0029, Fig. 2E).

**Fig. 3:**
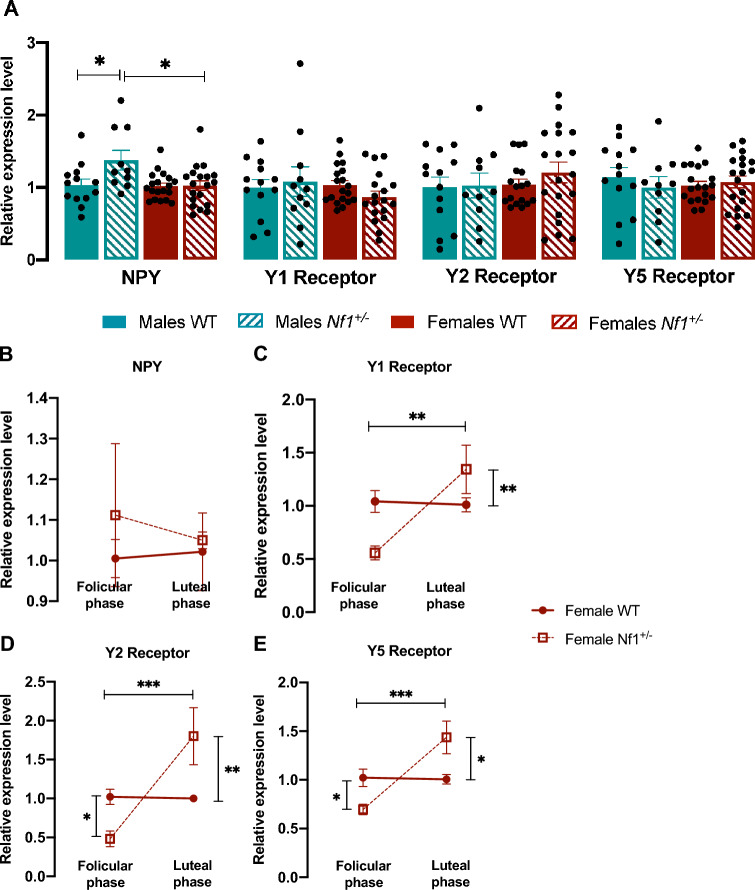
Hippocampal levels of the NPY system mRNA of male and female *Nf1*^+*/-*^ mice. (**A**) Relative mRNA expression of NPY and its receptors (Y1, Y2, and Y5) in the hippocampus of WT and *Nf1*^+*/-*^ males and females. Although no changes were observed in receptors, *NPY* levels were increased in mutant males comparing with *Nf1*^+*/-*^ females and WT littermates. Graphs depict individual values, mean, and standard error (n = 10 – 19 per group)). One-way ANOVA followed by Tukey’s post-hoc test was used for multiple comparisons .*p < 0.05. (**B**–**E**) mRNA expression of (**B**) *NPY*, (**C**) *Y1*, (**D**) *Y2*, and (**E**) *Y5* across the estrous cycle in *Nf1*^+*/-*^ females. Y1, Y2 and Y5 estrous cycle-dependent expression in mutant females, characterized by a downregulation during follicular phase and an upregulation during luteal phase. Graphs depict mean, and standard error (n = 6 – 3 per group). One-way ANOVA followed by Tukey’s post-hoc test was used for multiple comparisons *p < 0.05, **p < 0.01, ***p < 0.001; ****p < 0.0001.

On the other hand, the hippocampal data revealed greater relative *NPY* mRNA levels in male *Nf1*^+*/-*^ than in male WT littermate (Male WT: 1.037 ± 0.081, Male *Nf1*^+*/-*^*:* 1.376 ± 0.135, p = 0.035; Female *Nf1*^+*/-*^: 1.025 ± 0.066, p = 0.015, Fig. 3A). No additional alterations were observed (Fig. 3A). The hippocampal *NPY* gene expression of transgenic females did not change (Fig. 3B), but the receptors were differentially expressed according to the estrous cycle. Indeed, *Y1, Y2* and *Y5* mRNA were downregulated in the follicular phase and overexpressed in the luteal phase, suggesting that changes in the hippocampal NPY system may be estrous cycle-dependent (*Y1*—Female *Nf1*^+*/-*^ follicular phase: 0.558 ± 0.065, Female *Nf1*^+*/-*^ luteal phase: 1.343 ± 0.228, p = 0.0013, Fig. 3C; *Y2*—Female *Nf1*^+*/-*^ follicular phase: 0.482 ± 0.010, Female *Nf1*^+*/-*^ luteal phase: 1.800 ± 0.365, p < 0.0001, Fig. 3D; *Y5*—Female *Nf1*^+*/-*^ follicular phase: 0.696 ± 0.053, Female *Nf1*^+*/-*^ luteal phase: 1.437 ± 0.168, p = 0.0002, Fig. 3E). Furthermore, in general, the levels of *Y1, Y2* and *Y5* mRNAs were significantly different in both phases between *Nf1*^+*/-*^ and WT females (*Y1*—Female WT follicular phase: 1.042 ± 0.102, p = 0.0146, Fig. 3C; *Y2*—Female WT follicular phase: 1.022 ± 0.097, p = 0.0303; Female WT luteal phase: 1.002 ± 0.002, p = 0.0057, Fig. 3D; *Y5*—Female WT follicular phase: 1.023 ± 0.089, p = 0.044; Female WT luteal phase: 1.007 ± 0.050, p = 0.0208, Fig. 3E). However, no changes between the follicular and luteal phases were observed in WT females.

These results suggest a brain region- and sex-specific signature in the NPY system in a mouse model of ASD. We also found changes in the hippocampal and amygdalar NPY systems in females that were dependent on the estrous cycle; an interesting aspect requiring further validation.

### Nf1^+/-^ mice exhibit alterations in the intestinal NPY system

**Fig. 4:**
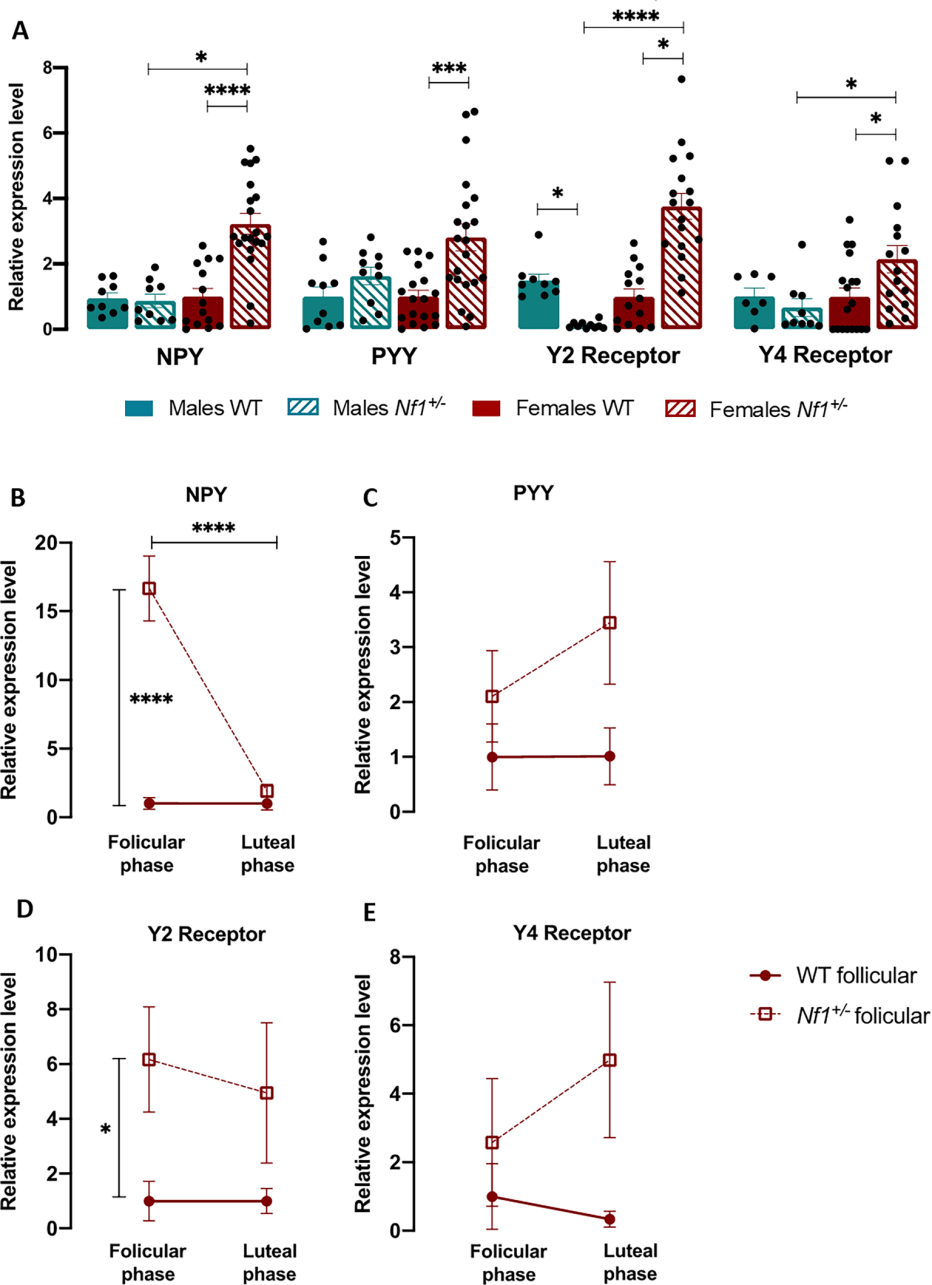
*Nf1*^+*/-*^mice exhibit sex-dependent changes in intestinal NPY system**.** (**A**) Relative expression of intestinal *NPY, PYY, Y2* and *Y4* mRNA. Transgenic females overexpressed *NPY, PYY*, *Y2* and *Y4* in the intestine. Mutant males only exhibited a decreased expression of *Y2.* Graphs depict individual values, mean, and standard error (n = 9 – 20 per group)). One-way ANOVA followed by Tukey’s post-hoc test was used for multiple comparisons *p < 0.05; ***p < 0.001; ****p < 0.0001. (**B**–**E**) mRNA expression of (**B**) *NPY*, (**C**) *Y1*, (**D**) *Y2*, and (**E**) *Y5* across the estrous cycle in *Nf1*^+*/-*^ females. Intestinal *NPY* and *Y2* expression was overexpressed only in the follicular phase of transgenic females. Graphs depict mean, and standard error (n = 6 – 3 per group)). One-way ANOVA followed by Tukey’s post-hoc test was used for multiple comparisons: *p < 0.05, ****p < 0.0001.

NPY is widely expressed not only in the central nervous system but also in the gastrointestinal tract. Furthermore, gastrointestinal dysfunction is associated with ASD pathogenesis and severity^40,41^. Investigating the intestinal mRNA levels of *NPY*, *PYY*, *Y2* and *Y4* (Fig. 4*)*, we observed that, compared with their WT littermates, only females *Nf1*^+*/-*^ presented an up-regulation mRNA levels (*NPY*—Female *Nf1*^+*/-*^: 3.226 ± 0.316, Female WT: 1.000 ± 0.252, p < 0.0001; *PYY*—Female *Nf1*^+*/-*^: 2.810 ± 0.414, Female WT: 1.000 ± 0.194, p = 0.0005; *Y2*—Female *Nf1*^+*/-*^: 3.759 ± 0.399, Female WT: 1.000 ± 0.236, p < 0.0001; *Y4*—Female *Nf1*^+*/-*^: 2.145 ± 0.415, Female WT: 0.999 ± 0.264, p = 0.0441; Fig. 4A). In some cases, also compared with transgenic males (*NPY*—Male *Nf1*^+*/-*^: 0.870 ± 0.210, p < 0.0001; *Y2*—Male *Nf1*^+*/-*^: 1.627 ± 0.167, p < 0.0001; *Y4*—Male *Nf1*^+*/-*^: 0.668 ± 0.271, p = 0.0277; Fig. 4A). Interestingly, intestinal *Y2* expression displays a sex signature, with *Nf1*^+*/-*^ males exhibited a decreased levels compared with sex-matched controls (Male *Nf1*^+*/-*^: 0.128 ± 0.033, Male WT: 1.495 ± 0.188, p = 0.046; Fig. 4A). Importantly, these sex-dependent alterations were not observed in control animals.

When we analyzed the expression of *NPY, PYY*, and their receptors *Y2* and *Y4* concerning the estrous cycle, we detected that only *NPY* mRNA expression showed cycle-dependent changes in *Nf1*^+*/-*^ animals (*NPY*—Female *Nf1*^+*/-*^ follicular phase: 16.66 ± 2.371, Female *Nf1*^+*/-*^ luteal phase: 1.909 ± 0.475, p < 0.0001, Fig. 4B). Moreover, in the follicular phase, the mutated female had a significantly increase in *NPY* and *Y2* levels comparing with their littermates WT, which was not observed in the luteal phase (*NPY*—Female WT follicular phase: 1.000 ± 0.421, p < 0.0001, Fig. 4B; *Y2*—Female WT follicular phase: 1.000 ± 0.724, p = 0.0433, Fig. 4D). Overall, our data indicate that, similar to that in the brain, the intestinal NPY system might be altered in an animal model of ASD in a sex-dependent manner.

**Fig. 5:**
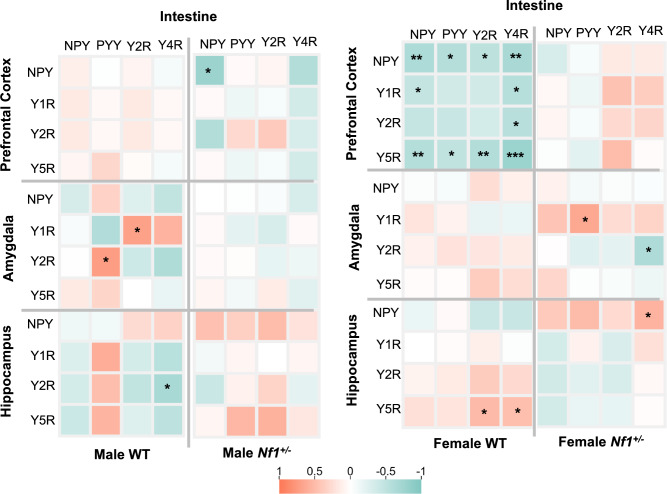
*Nf1*^+*/-*^ females lose correlation between intestine and central NPY system. (**A**, **B**) Heatmap illustrating differential correlation data between intestine-brain in both (**A**) males and (**B**) females of control and transgenic mice. Correlation heatmap matrix were performed using Spearman’s analysis of absolute values of ΔCq. Significance levels are as follows: * p < 0.05, ** p < 0.01, *** p < 0.001.

To evaluate the correlation between central and intestinal NPY system changes, we performed an exploratory correlation analysis. The results suggest that the central NPY system is associated with intestinal NPYergic signaling (Fig. 5), especially in females. Interestingly, WT male mice had a positive correlation between amygdalar *Y1* and intestinal *Y2* (Spearman r = 0.700, p = 0.043) and amygdalar *Y2* and intestinal *PYY* (Spearman r = 0.683, p = 0.040), which was absent in mutant animals (Fig. 5A). Other minor observation included a negative correlation between cortical and intestine *NPY* observed only in transgenic males (Spearman r = −0.786, p = 0.028; Fig. 5A). Concerning females, we found that WT mice display a global negative correlation between cortical and intestinal NPY system (cortical NPY – intestinal NPY: Spearman r = -0.649, p = 0.004; cortical NPY – intestinal PYY: Spearman r = -0.589, p = 0.010; cortical NPY – intestinal Y2: Spearman r = -0.525, p = 0.025; cortical NPY – intestinal Y4: Spearman r = -0.676, p = 0.002; cortical Y5 – intestinal NPY: Spearman r = -0.598, p = 0.009; cortical Y5 – intestinal PYY: Spearman r = -0.492, p = 0.038; cortical Y5 – intestinal Y2: Spearman r = -0.614, p = 0.007; cortical Y5 – intestinal Y4: Spearman r = -0.794, p < 0.0001; cortical Y1 – intestinal Y4: Spearman r = -0.579, p = 0.012; cortical Y2 – intestinal Y4: Spearman r = -0.566, p = 0.014; Fig. 5B). However, all these correlations were lost in *Nf1*^+*/-*^ female mice (Fig. 5B). Other minor alterations were: *Nf1*^+*/-*^ females only: amygdalar *Y2* –intestinal *Y4*: Spearman r = -0.642, p = 0.015; amygdalar *Y1* – intestinal *PYY:* Spearman r = 0.653, p = 0.013; hippocampal *NPY* – intestinal *Y4:* Spearman r = 0.570, p = 0.036; WT females only: hippocampal *Y5* – intestinal *Y2*: Spearman r = 0.514, p = 0.029, and *Y4*: Spearman r = 0.478, p = 0.045; Fig. 5B). Overall, results suggest that changes in the central NPY system are closely linked to NPYergic intestinal changes.

### Intestinal dysbiosis in female Nf1^+/-^ mice is associated with the intestinal NPY system

**Fig. 6:**
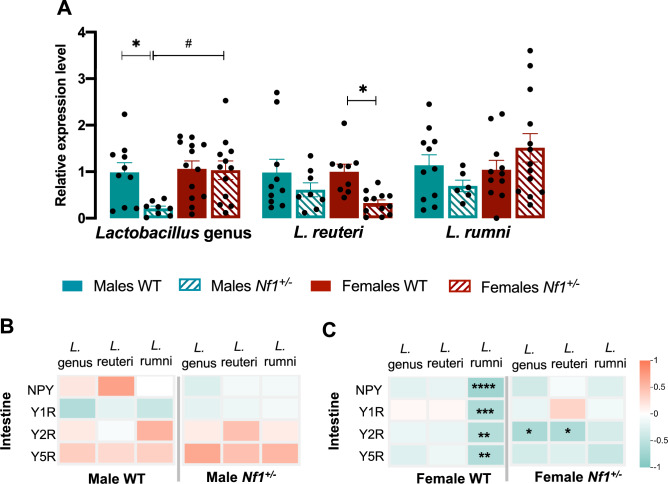
Dysbiosis in female *Nf1*^+*/-*^ mice is strongly correlated with intestinal NPY system. (**A**) Characterization of the relative amount of genus Lactobacillus and species in the gut stool. Transgenic males displayed a reduction in the relative content of Lactobacillus. No alterations were observed in the females’ group. L reuteri levels were only reduced in the stool of *Nf1*^+*/-*^ females. Concerning *L. rumni*, no changes were detected among the groups. (**B**, **C**) Heatmap illustrating differential correlation data between intestine-gut microbiota in both (**B**) males and (**C**) females of control and transgenic mice. Correlation heatmap matrix were performed using Spearman’s analysis of absolute values of ΔCq. Significance levels are as follows: * p < 0.05, ** p < 0.01, *** p < 0.001.

A recent study demonstrated that the NPYergic system modulated the microbial diversity of the gut microbiome^42^. We thus examined whether the gut microbiome of *Nf1*^+*/-*^ mice was altered, with a focus on the on Lactobacillus genus and species because this genus has previously been widely implicated in ASD^17,43–45^. At the genus level, we specifically found that transgenic males exhibit a reduction in the relative abundance of the Lactobacillus genus to their WT littermates and female *Nf1*^+*/-*^ (Male *Nf1*^+*/-*^: 0.210 ± 0.052, Male WT: 0.985 ± 0.209, p = 0.0435; Female *Nf1*^+*/-*^: 1.031 ± 0.199, p = 0.022; Fig. 6A). No significant differences were found between *Nf1*^+*/-*^ and WT littermate females. Next, we investigated the changes in selected species of Lactobacillus, namely *Limosilactobacillus reuteri (L.reuteri) and Lactobacillus rumni (L.rumni).* Althought no significant changes were observed between males, *Nf1*^+*/-*^ females display decreased levels of *L. reuteri*, suggesting a sex-specific dysregulation (Female *Nf1*^+*/-*^: 0.325 ± 0.069, Female WT: 1.000 ± 0.162, p = 0.0460; Fig. 6A*)*. Concerning *L. rumni*, no significant changes were observed among groups (Fig. 6A*).* We did not detect significant differences in the segregation of females for the estrous cycle, indicating that the female cycle might not interfere with *Lactobacillus* genus alterations (see Additional file 2).

To understand whether alterations in the *Lactobacillus* genus and species are associated with intestinal NPY system, an exploratory correlation analysis was performed (Fig. 6B,C). No correlations were found in both *Nf1*^+*/-*^ and WT males (Fig. 6B). Contrary, in WT females, *Lactobacillus* abundance, especially *L. rumni*, was negatively correlated with intestinal *NPY (*Spearman r = -0.761, p < 0.0001)*, PYY (*Spearman r = -0.641, p = 0.004)*, Y2* (Spearman r = -0.589, p = 0.006) and *Y4* (Spearman r = -0.620, p = 0.010), Fig. 6C. Mutated females lost *L.rumni-*intestine NPYergic correlations, though negative correlations were observed between intestinal *Y2* and Lactobacillus (Spearman r = -0.670, p = 0.011) and *L.reuteri* (Spearman r = −0.644, p = 0.015; Fig. 6C). To summarize, *Nf1*^+*/-*^ animals showed alterations in Lactobacillus genus, some of which were associated with intestinal NPYergic system in females.

### Transgenic females are more susceptible to molecular changes in NPY gut-brain communication and the microbiota

**Fig. 7:**
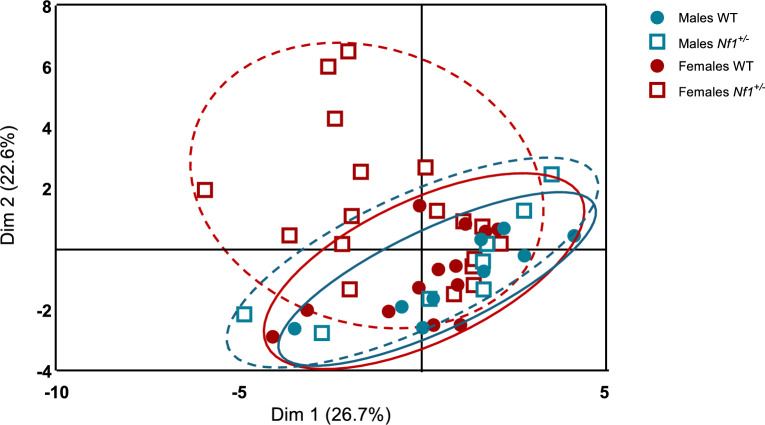
Transgenic females exhibit a different molecular pattern of NPY along the microbiota-gut-brain-system. Transgenic females display a distinct populational cluster in PCA analysis. Populational clusters were obtained for wildtypes and transgenic from both sexes based on NPY-gut-brain expression and microbiota abundance. Percentage values in parentheses next to dimension 1 (Dim1) and dimension 2 (Dim2) represent the percentage of variance explained by each component.

To better understand the overall transcriptomic effect between the intestinal and brain NPYergic system and the microbiota, PCA analysis was performed. Briefly, PCA is a technique that transforms a dataset with interrelated or correlated variables into a new dataset with uncorrelated variables known as principal components (PCs) which helps in describing multivariate patterns and variation in behavioral outcomes across groups. The results of the PCA revealed that transgenic females display a distinct cluster based on NPY-gut-brain transcriptomic data, emphasizing the distinct molecular profile in NPY in microbiota-gut-brain of transgenic females (Fig. 7). The loadings of variables and their relative importance for PCA were extrapolated when comparing transgenic and WT. Across all groups, the variables that primarily contributed to explaining individual variance were particularly correlated with NPYergic expression in PFC, amygdala, and intestine, which were affected particularly in mutant females based on our results (Additional files – Additional Fig. 3).

## Discussion

Our study provides the first evidence that the NPY system is altered in the gut-brain axis of a mouse model of ASD. These changes were mainly observed in female *Nf1*^+*/-*^ mice and some were dependent on the estrous cycle. Interestingly, in females, we also found significant correlations between the intestinal and central NPYergic systems, as well as with *Lactobacillus* levels. These findings led us to hypothesize that the NPY system may play a crucial role in maintaining gut homeostasis.

NPY has been identified as a possible neurochemical mediator for the interaction of the gut-microbiota with brain function^22^. It was reported that the brain NPY system is severely disturbed by antibiotic-induced gut dysbiosis, namely in the hippocampus, amygdala and hypothalamus of adult male C57BL/6N mice^46^. More recently, another study using a model of postmenopausal osteoporosis showed NPY and Y1 modulated the microbial diversity and changed the community composition of the gut microbiome in ovariectomized female rats^42^. However, studies associating the NPYergic system with the changes observed in the gut-brain axis in individuals with autism are still lacking. Moreover, given the well-documented sexual dimorphisms in this system^47^, investigating how NPYergic alterations differ between males and females is crucial.

Our data suggests sex-dependent changes in the brain NPYergic system in *Nf1*^+*/-*^ animals. While the main alterations in the NPY system were observed in the cortex and amygdala of transgenic females, changes in the hippocampus were exclusive to the *Nf1*^+*/-*^ males. These findings support the hypothesis that male *Nf1*^+*/-*^ mice are more prone to develop cognitive impairments, as reported by our group^9^. Under physiological conditions, central NPY plays a crucial role in modulating neuroplasticity, neurotransmission, and memory^48^ by regulating synaptic plasticity and cognitive functions^49^. Accordingly, under pathological conditions, treatment with NPY system modulators has been shown to reverse cognitive impairment^42,50,51^. Our previous study demonstrated that *Nf1*^+*/−*^ females exhibited more severe anxiety-related traits^9^, which may be influenced by alterations in the cortical and amygdalar NPYergic system. The observed reduction in cortical NPY levels and increased Y2 receptor expression in the amygdala align with evidence suggesting that these molecular changes are associated with an enhanced anxiogenic phenotype^23,24,26,52,53^. Notably, reduced NPY levels have been consistently reported in both preclinical and clinical studies of depression, chronic stress, and post-traumatic stress disorder^54–56^. Furthermore, *postmortem* studies have revealed a significant decrease in *NPY* mRNA, along with an upregulation in *Y2* mRNA in the hippocampus and PFC regions of individuals with depression and suicide behaviors^57^. Recent findings by Carbona et al. highlight important sex-dependent differences in NPY levels in the PFC of young juvenile rats, which are linked to anxiety-related behavior^58^. Although few studies have directly examined the causal relationship between Y2 in the amygdala and anxiogenesis, evidence suggests that activation of Y2 receptors, which are predominantly expressed on presynaptic terminals in the basolateral amygdala, can exacerbate anxiety behavior^23^. Based on these findings, we propose that dysfunction in the brain NPY system represents an adaptive functional response to counterbalance the behavioral impairments observed in *Nf1*^+*/-*^ animals in a sex-dependent manner^9^.

Previous studies have suggested that the estrous cycle influences the expression of anxiety- and autism-like behaviors in rodents^59,60^. Moreover, other authors reported that fluctuations in circulating levels of estrogen and progesterone, depending by estrous cycle, are paralleled by changes in the expression of central NPY system^61,62^. Our findings align with this evidence. In our mouse model of ASD, we observed that the NPY system in the amygdala and hippocampus—two regions that work together to regulate the hypothalamic–pituitary–adrenal (HPA) axis—is influenced by the estrous cycle^63^. Notably, the most significant alterations in the amygdala occurred during the luteal phase, which coincided with the lowest levels of gonadal hormones. We hypothesize that the increase in NPY system levels during the luteal phase may be linked to the modulation of anxiety-related behavior. This is supported by findings from Lebron-Milad & Milad, who reported that females in the luteal phase exhibit greater anxiety-like behavior than do those in the follicular phase or males^64^. Interestingly, compared with those in WT females, NPY receptor levels in the hippocampus are downregulated during the follicular phase and upregulated during the luteal phase. The NPY system is a well-established neuromodulator widely distributed in the hippocampus^65^ playing a crucial role in cognitive processing^66^. Our findings are in agreement with recent advancements in multiphoton imaging techniques, which highlight the estrous cycle as a driver of large-scale structural and functional plasticity in the hippocampal circuits essential for learning and memory^67^.

The NPY system may act as a neurochemical mediator in the gut-brain axis^22^, and our findings support this hypothesis. In addition to alterations in the central NPY system, we also observed changes in the intestinal NPY system, which were once again sex-dependent. Our study provides evidence that females *Nf1*^+*/-*^ mice are particularly susceptible to transcriptomic changes in intestinal *NPY, PYY,* and their receptor Y4. Interestingly, the expression patterns of intestinal *Y2* differed between males and females. While *Nf1*^+*/-*^ males showed a significant reduction in Y2 expression, mutant females showed an increase. Notably, these alterations were not influenced by the estrous cycle. Previously, our group have demonstrated that female *Nf1*^+*/-*^ mice show surprisingly better spatial memory performance, in contrast to the severe cognitive impairments observed in male *Nf1*^+*/-*^ mice^9^. Accordingly, gastrointestinal issues have been correlated with poorer cognitive performance in ASD cases^68^. Furthermore, the higher prevalence of ASD in boys may be linked to distinct changes in the intestinal microbiome compared to affected girls^69^. On the other hand, given that both autistic female^70–72^ and *Nf1*^+*/-*^ females^9^ are more prone to developing, our results provide evidence that NPY signaling could be a promising pathway involved in intestinal-cognitive dysfunction in individuals with ASD. Consistent with this hypothesis, we found that some correlations between the cortical and hippocampal NPY systems and the intestinal NPY system were lost in mutant females, while new correlations were observed in the amygdala. Overall, we suggest that sex-biased dysfunction of the NPYergic system could provide an important framework for understanding gut-brain abnormalities in individuals with ASD.

Impaired gastrointestinal function is accompanied by an imbalance in the composition of the microbiota^73^. Biological sex is also thought to play a prominent role in the sexual dimorphism of gut microbiota^18,44,74^. Here, we have also reported a sex-dependent dysbiosis in the *Lactobacillus* genus and *L. reuteri* in *Nf1*^+*/-*^ animal model. We found that transgenic females exhibited decreased levels of *L. reuteri*, whereas males presented decreased levels of the *Lactobacillus* genus. Accordingly, Tabouy et al. found that dysregulation of *Lactobacillus* and *L.reuteri* in the gut of both males and females of *Shank3*^*-/-*^ mice is associated with social impairments and stereotypic-like behaviors^17^. Furthermore, another study found that administration of a specific strain *L. reuteri* ameliorated social deficits in several ASD mouse models^44^. While this targeted approach allowed us to investigate a genetically relevant and functionally important bacterial genus, we acknowledge that PCR-based profiling provides a limited view of the gut microbiome. In addition to confirming these changes in the *Lactobacillus* genus, we found an association between dysbiosis and the intestinal NPYergic system. The negative correlations found in WT females between the intestinal NPYergic system and *L. rumni* were lost in *Nf1*^+*/-*^ females, which in turn, developed a new significant correlation between *L. reuteri* and intestinal *Y2*. No changes were detected in males. This finding leads us to postulate that an increase in intestinal *Y2* could be a strategy to minimize to negative effect of dysbiosis, at least in ASD females. Previous studies hypothesized that restoring levels of *L. reuteri* resulted in higher levels of oxytocin, a hormone associated with social behavior^17,18,44^. With our results, we provide new evidence that *L. reuteri* could model other peptide systems at the level of the gut-brain axis, namely the NPY system. In fact, recently, new finding exploring the influence of gut microbiota on NPY expression in the colon and the concomitant alteration in the NPY of hippocampus, pointed out a microbiome-based therapy to precisely adjust the expression and function of NPY^75^. Therefore, understanding the intricate interplay between gut microbiota and NPY holds great promise for improving the holistic care of individuals with neurodevelopmental disorders.

## Limitations

Despite the significant findings of this study, several important considerations remain. First, to generalize these conclusions, it is essential to examine additional ASD mutant mouse models, particularly focusing on NPY expression as explored in this study. Different ASD mouse models may exhibit varying patterns of NPY expression in the colon and key brain regions involved in social behavior, such as the amygdala, hippocampus, and prefrontal cortex, with potential differences based on biological sex. Second, while PCR is a powerful tool for quantifying the relative abundance of gut microbial species, its effectiveness is limited by the small number of genera and species analyzed. To achieve a more comprehensive understanding of gut microbiota composition, future research should integrate complementary techniques that offer greater sensitivity and resolution. Further, our study reports transcriptional changes; it is important to note that mRNA alterations do not always directly translate into protein-level changes due to post-transcriptional regulation and protein stability. Third, given the well-established influence of the menstrual cycle on female physiology, increasing the sample size could provide clearer insights into the role of menstrual phases in ASD pathophysiology through an NPY-dependent mechanism. Lastly, the relationship between alterations in the gut-brain NPY system and microbiota dysbiosis remains unclear. To address this gap, targeted microbiota-based interventions, such as probiotic supplementation or fecal microbiota transplants, should be explored as potential strategies for precisely modulating the gut microbiome and elucidating its impact on the gut-brain axis. Finally, the correlation analyses were conducted with an exploratory, hypothesis-generating aim, which constitutes an important limitation worth highlighting. Consequently, the observed associations should be interpreted with caution, as they may reflect spurious or non-causal relationships.

## Conclusion

Our work suggests for the first time that the NPYergic receptor system may represent an underlying molecular contributor to the association between gastrointestinal dysfunction and the brain pathophysiology of ASD. The interaction between the brain and gut was influenced by both sex and specific regions. Female ASD mice appear to be more vulnerable to NPYergic changes in both the gut and brain, and some changes seem to be modulated by gonadal hormones during the estrous cycle. The intestinal Y2 receptor has emerged as a promising biomarker for understanding ASD-related gut-brain dysfunction. These alterations in the NPY system were also correlated with microbiome dysbiosis, a key characteristic observed in individuals with autism. Collectively, these findings are important for advancing our understanding of atypical development in patients with neuropsychiatric disorders such as ASD. These findings open new avenues for exploring sex-dependent interactions between the brain, gut, and microbiome, with the NPYergic system identified as a critical player in this relationship.

## Supplementary Information

Supplementary Information.

## Data Availability

The datasets supporting the conclusions of this article are included within the article and its additional files.

## Abbreviations

ASD: Autism spectrum disorder
mRNA: Messenger ribonucleic acid
NF1: Neurofibromatosis type 1
NPY: Neuropeptide Y
PCA: Principal component analysis
PCR: Polymerase chain reaction
WT: Wild-type
