# Supplementary material for: Sex-dependent dysregulation of the gut-brain NPYergic system in a mouse model of autism spectrum disorder
Source: Sci Rep. 2026 Mar 4;16:11931. doi: 10.1038/s41598-026-42601-0 (PMC13068916; doi:10.1038/s41598-026-42601-0)
Supplement: Supplementary file 1 — Supplementary Information. [file 41598_2026_42601_MOESM1_ESM.docx]

**Additional files**

**Table 1**: Primers used for qRT-PCR

| Category | Target | Primer direction | Primer sequence (5’-3’) |
| --- | --- | --- | --- |
| Neuropeptide Y | *NPY* | FWD  REV | CACCAGACAGAGATATGGCAAGA  TGTTCTGGGGGCGTTTTCTG |
|  | *Y1* | FWD  REV | CCCATCTGACTCTCACAGGC  AGCGAATGTATATCTTGAAGTAGCA |
|  | *Y2* | FWD  REV | CGCAAGAGTCAATACAGCCAA  CCCATAGGGCTCCACTTTCA |
|  | *Y4* | FWD  REV | TGTGCCTCATCTGCCAACCA  CTCTCCAGGGCCACAAGGAC |
|  | *Y5* | FWD  REV | TTCCATCTCAAGCAGAAGCGA  CATACTAGAGTCCTCGGGATGC |
|  | *PYY* | FWD  REV | CCGGCAGCGGTATGGAAAAA  TTCTGGCCTGAAGGGGAGG |
| Housekeeping | *B2M* | FWD  REV | CATGGCTCGCTCGGTGAC  CAGTTCAGTATGTTCGGCTTCC |
|  | *YWHAZ* | FWD  REV | GAAAAGTTCTTGATCCCCAATGC  TGTGACTGGTCCACAATTCCTT |
|  | *HRPT* | FWD  REV | GCTTACCTCACTGCTTTCCG  CATCATCGCTAATCACGACGC |
| Microbiome | *Lactobacillus* | FWD  REV | GAGGCAGCAGTAGGGAATCTT  GGCCAGTTACTACCTCTATCCTTCTTC |
|  | *L. reuteri* | FWD  REV | GAAGATCAGTCGCAYTGGCCCAA  TCCATTGTGGCCGATCAG |
|  | *L. rumni* | FWD  REV | CACCGAATGCTTGCAYTCACC  GCCGCGGGTCCATCCAAAA |
|  | *16S* | FWD  REV | ACGTCRTCCMCNCCTTCCTC  GTGSTGCAYGGYYGTCGTCA |

**Table 2**

*NPY* and receptors expression in CNS

|  | **Gene** | **Genotype** | **Sex** | **N** | **Mean Value**  **± SEM** | **95 % CI of mean** | **P-value** |
| --- | --- | --- | --- | --- | --- | --- | --- |
| **Prefrontal cortex** | *NPY* | WT | M | 12 | 1.000±0.0929 | 0.8415-1.250 | p=0.9665 |
|  |  | *Nf1^+/-^* |  | 9 | 0.9378±0.048 | 0.8276-1.048 |  |
|  |  | WT | F | 19 | 1.242±0.1934 | 0.8358-1.648 | ****p=0.0084** |
|  |  | *Nf1^+/-^* |  | 16 | 0.6450±0.041 | 0.5569-0.7331 |  |
|  | *Y1* | WT | M | 12 | 1.000±0.1346 | 0.7037-1.296 | p=0.4873 |
|  |  | *Nf1^+/-^* |  | 9 | 0.7404±0.099 | 0.5114-0.9695 |  |
|  |  | WT | F | 19 | 0.999±0.122 | 0.7435-1.256 | ***p=0.0358** |
|  |  | *Nf1^+/-^* |  | 17 | 0.6162±0.054 | 0.5010-0.7315 |  |
|  | *Y2* | WT *Nf1^+/-^* | M | 12  9 | 1.000±0.1698  0.6786±0.0099 | 0.6266-1.374  0.4498-0.9075 | p=0.4166 |
|  |  | WT  *Nf1^+/-^* | F | 18  17 | 0.999±0.1378  0.7138±0.067 | 0.7091-1.290  0.5722-0.8553 | p=0.2870 |
|  | *Y5* | WT  *Nf1^+/-^* | M | 13  9 | 1.00±0.09615  0.5712±0.065 | 0.7907-1.210  0.4205-0.7220 | p=0.3348 |
|  |  | WT  *Nf1^+/-^* | F | 18  17 | 0.990±0.2011  0.4562±0.054 | 0.5756-1.424  0.3422-0.5702 | ***p=0.0194** |
| **Hippocampus** | *NPY* | WT  *Nf1^+/-^* | M | 13  10 | 1.00±0.081  1.376±0.1348 | 0.8614-1.212  1.071-1.681 | ***p=0.0350** |
|  |  | WT  *Nf1^+/-^* | F | 19  19 | 1.015±0.043  1.025±0.066 | 0.9252-1.105  0.8866-1.163 | p=0.9996 **^#^p=0.0150** |
|  | *Y1* | WT  *Nf1^+/-^* | M | 13  11 | 1.00±0.1105  1.081±0.2052 | 0.7592-1.241  0.6236-1.538 | p=0.9646 |
|  |  | WT  *Nf1^+/-^* | F | 19  18 | 1.033±0.062  0.8672±0.082 | 0.9018-1.163  0.6952-1.039 | p=0.6250 |
|  | *Y2* | WT  *Nf1^+/-^* | M | 13  10 | 1.000±0.1413  1.028±0.1704 | 0.6922-1.308  0.6421-1.413 | p=0.9992 |
|  |  | WT  *Nf1^+/-^* | F | 17  19 | 1.039±0.075  1.207±0.1438 | 0.8802-1.199  0.9047-1.509 | p=0.7600 |
|  | *Y5* | WT  *Nf1^+/-^* | M | 13  10 | 0.9674±0.115  0.8466±0.1241 | 07169-1.218  0.5659-1.127 | p=0.8434 |
|  |  | WT  *Nf1^+/-^* | F | 19  19 | 1.026±0.0555  1.078±0.0845 | 0.9092-1.142  0.9004-1.255 | p=0.9673 |
| **Amygdala** | *NPY* | WT | M | 13 | 1.000±0.0915 | 0.9335-1.155 | p=0.9929 |
|  |  | *Nf1^+/-^* |  | 10 | 1.087±0.1497 | 0.915-1.228 |  |
|  |  | WT | F | 20 | 1.070±0.0707 | 0.9221-1.218 | p=0.9812 |
|  |  | *Nf1^+/-^* |  | 12 | 1.093±0.0740 | 0.9306-1.256 |  |
|  | *Y1* | WT | M | 13 | 1.053±0.1031 | 0.8281-1.278 | p=0.9777 |
|  |  | *Nf1^+/-^* |  | 8 | 1.105±0.1193 | 0.8229-1.387 |  |
|  |  | WT | F | 20 | 1.000±0.0058 | 0.9177-1.130 | p=0.9656 |
|  |  | *Nf1^+/-^* |  | 14 | 0.9771±0.0649 | 0.8369-1.117 |  |
|  | *Y2* | WT  *Nf1^+/-^* | M | 12 | 1.000±0.05079 | 0.9249-1.148 | p=0.5301 |
|  |  |  |  | 10 | 0.8730±0.0543 | 0.7502-0.9958 |  |
|  |  | WT  *Nf1^+/-^* | F | 19 | 1.056±0.8126 | 0.8856-1.227 | ***p=0.0277 ^##^p=0.0010** |
|  |  |  |  | 14 | 1.343±0.0801 | 1.170-1.516 |  |
|  | *Y5* | WT  *Nf1^+/-^* | M | 13 | 1.000±0.07725 | 0.8632-1.200  0.7333-1.287 | p=0.9970 |
|  |  |  |  | 10 | 1.010±0.1223 |  |  |
|  |  | WT  *Nf1^+/-^* | F | 19 | 1.013±0.0389 | 0.9315-1.095  1.035-1.276 | p=0.4519 |
|  |  |  |  | 11 | 1.155±0.0542 |  |  |

Mean values of normalized gene expression, 95% confidence intervals and P values obtained by Two-way ANOVA with Tukey's post hoc correction analysis for both genotypes according to each sex. CI: Confidence Interval; F: Female; M: Male; N: Sample size; SEM: Standard Error of the Mean
* (*Nf1^+/-^* versus WT in the same sex) p<0.05; **p<0.001; ***p<0.0001; ^#^(Male *Nf1^+/-^* versus Female *Nf1^+/-^*)<0.0.5; ^##^p<0.001; ^###^p<0.0001;

**Table 3**

Female central *NPY* expression according to ovulatory cycle.

|  | **Gene** | **Genotype** | **Estrous**  **cycle** | **N** | **Mean Value**  **± SEM** | **95 % CI of mean** | **P-value** |
| --- | --- | --- | --- | --- | --- | --- | --- |
| **Prefrontal cortex** | *NPY* | WT | Follicular | 6 | 1.000±0.2386 | 0.3866-1.613 | p=0.6081 |
|  |  | *Nf1^+/-^* |  | 5 | 0.974±0.1287 | 0.6168-1.331 |  |
|  |  | WT | Luteal | 6 | 1.086±0.2807 | 0.3643-1.808 | p=0.9953 |
|  |  | *Nf1^+/-^* |  | 3 | 1.208±0.1895 | 0.6818-1.734 |  |
|  | *Y1* | WT | Follicular | 6 | 1.000±0.1712 | 0.6432-1.523 | p=0.3793 |
|  |  | *Nf1^+/-^* |  | 5 | 0.6050±0.088 | 0.3247-0.8853 |  |
|  |  | WT | Luteal | 6 | 1.135±0.2593 | 0.4685-1.801 | p=0.9987 |
|  |  | *Nf1^+/-^* |  | 3 | 1.087±0.04910 | 0.8754-1.298 |  |
|  | *Y2* | WT *Nf1^+/-^* | Follicular | 6  5 | 1.000±0.2716  0.4906±0.0782 | 0.2460-1.754  0.2734-0.7077 | p=0.3229 |
|  |  | WT  *Nf1^+/-^* | Luteal | 6  3 | 0.9663±0.2107  0.6804±0.1650 | 0.4247-1.508  -0.02952-1.390 | p=0.8098 |
|  | *Y5* | WT  *Nf1^+/-^* | Follicular | 6  5 | 1.000±0.3886  0.7361±0.4897 | 0.00098-1.999  -0.6236-2.096 | p=0.9578 |
|  |  | WT  *Nf1^+/-^* | Luteal | 6  3 | 0.9873±0.2643  0.7986±0.3425 | 0.3080-1.667  -0.6752-2.272 | p=0.9896 |
| **Hippocampus** | *NPY* | WT  *Nf1^+/-^* | Follicular | 6  5 | 1.005±0.04667  1.112±0.1758 | 0.8850-1.125 0.6239-1.600 | p=0.8871 |
|  |  | WT  *Nf1^+/-^* | Luteal | 6  3 | 1.022±0.09548  1.050±0.02082 | 0.7762-1.267  0.9604-1.140 | p=0.9984 |
|  | *Y1* | WT  *Nf1^+/-^* | Follicular | 6  5 | 1.042±0.1019  0.5580±0.0648 | 0.7797-1.304  0.3782-0.7378 | ***p=0.0146** |
|  |  | WT  *Nf1^+/-^* | Luteal | 6  3 | 1.010±0.06653  1.343±0.2282 | 0.8390-1.181  0.3614-2.325 | p=0.2093 **^##^p=0.0013** |
|  | *Y2* | WT  *Nf1^+/-^* | Follicular | 6  5 | 1.022±0.09748  0.4820±0.0999 | 0.7711-1.272  0.2046-0.7594 | ***p=0.0303** |
|  |  | WT  *Nf1^+/-^* | Luteal | 6  3 | 1.000±0.02798  1.800±0.3650 | 0.9297-1.074  0.2297-3.370 | ****p=0.0057 ^####^p<0.0001** |
|  | *Y5* | WT  *Nf1^+/-^* | Follicular | 6  5 | 1.020±0.08987  0.6960±0.0531 | 0.7907-1.253  0.5487-0.8433 | ***p=0.0441** |
|  |  | WT  *Nf1^+/-^* | Luteal | 6  3 | 1.000±0.04951  1.437±0.1676 | 0.8794-1.134  0.7157-2.158 | ***p=0.0208 ^###^p<0.0002** |
| **Amygdala** | *NPY* | WT | Follicular | 6 | 1.017±0.0765 | 0.8200-1.213 | ***p=0.0366** |
|  |  | *Nf1^+/-^* |  | 5 | 0.9740±0.1287 | 0.6168-1.331 |  |
|  |  | WT | Luteal | 6 | 1.055±0.1715 | 0.6141-1.496 | p=0.9975 **#p=0.0245** |
|  |  | *Nf1^+/-^* |  | 3 | 1.847±0.3666 | 0.2692-3.424 |  |
|  | *Y1* | WT | Follicular | 6 | 1.012±0.07378 | 0.8220-1.201 | p=0.4731 |
|  |  | *Nf1^+/-^* |  | 5 | 0.888±0.02746 | 0.8118-0.9642 |  |
|  |  | WT | Luteal | 6 | 1.000±0.050 | 0.8802-1.136 | ****p=0.0054 ###p=0.0006** |
|  |  | *Nf1^+/-^* |  | 3 | 1.397±0.09404 | 0.9920-1.801 |  |
|  | *Y2* | WT  *Nf1^+/-^* | Follicular | 6 | 1.107±0.1954 | 0.6044-1.609 | p=0.9672 |
|  |  |  |  | 5 | 1.208±0.1895 | 0.818-1.734 |  |
|  |  | WT  *Nf1^+/-^* | Luteal | 6 | 1.022±0.095 | 0.7777-1.266 | p=0.6862 |
|  |  |  |  | 3 | 1.310±0.038 | 1.147-1.473 |  |
|  | *Y5* | WT  *Nf1^+/-^* | Follicular | 6 | 1.012±0.0724 | 0.8255-1.198  0.8301-1.582 | p=0.4874 |
|  |  |  |  | 5 | 1.206±0.1354 |  |  |
|  |  | WT  *Nf1^+/-^* | Luteal | 6 | 1.000±0.02324 | 0.9403-1.060  0.8062-2.534 | ****p=0.0029 #p=0.0487** |
|  |  |  |  | 3 | 1.670±0.2007 |  |  |

Mean values of normalized gene expression, 95% confidence intervals and respective P values obtained by Two-way ANOVA with Tukey's post hoc comparisons-test for both genotypes according to each estrous cycle. CI: Confidence Interval; N: Sample size; SEM: Standard Error of the Mean
* (*Nf1^+/-^* versus WT in the same estrous cycle) p<0.05; **p<0.001; ***p<0.0001; ^#^(Luteal phase *Nf1^+/-^* versus Follicular *Nf1^+/-^*)<0.0.5; ^##^p<0.001; ^###^p<0.0001;

**Table 4**

*NPY* and *PYY* and receptors expression in colonic intestinal portion and microbiome relative abundance.

|  |  | **Genotype** | **Sex** | **N** | **Mean Value**  **± SEM** | **95 % CI of mean** | **P-value** |
| --- | --- | --- | --- | --- | --- | --- | --- |
| **Intestine** | *NPY* | WT | M | 10 | 0.9320±0.1441 | 0.6060-1.258 | p=0.9984 |
|  |  | *Nf1^+/-^* |  | 9 | 0.8700±0.2092 | 0.3875-1.353 |  |
|  |  | WT | F | 14 | 1.000±0.2516 | 0.4566-1.544 | ******p<0.0001 ^####^p<0.0001** |
|  |  | *Nf1^+/-^* |  | 20 | 3.226±0.2516 | 2.564-3.887 |  |
|  | *PYY* | WT | M | 10 | 1.000±0.2998 | 0.3219-1.678 | p=0.7207 |
|  |  | *Nf1^+/-^* |  | 10 | 1.627±0.2673 | 1.022-2.231 |  |
|  |  | WT | F | 18 | 1.030±0.1940 | 0.5909-1.410 | *****p<0.0005** |
|  |  | *Nf1^+/-^* |  | 21 | 2.810±0.4136 | 1.948-3.673 |  |
|  | *Y2* | WT  *Nf1^+/-^* | M | 10  10 | 1.544±0.1752  0.1276±0.0333 | 1.147-1.940 0.05215-0.2031 | *** p=0.0463** |
|  |  | WT  *Nf1^+/-^* | F | 18  21 | 1.000±0.2364 3.759±0.3984 | 0.4891-1.510 2.914-4.604 | ******p<0.0001 ^####^p<0.0001** |
|  | *Y4* | WT  *Nf1^+/-^* | M | 7  9 | 1.011±0.2424  0.6678±0.2715 | 0.4183-1.605 0.04166-1.294 | p=0.9416 |
|  |  | WT  *Nf1^+/-^* | F | 18  15 | 1.000±0.2637  2.145±0.4154 | 0,4433-1,556 1.254-3.037 | ***p=0.0441**  **^#^p=0.0277** |
| **Microbiome** | Lactobacillus | WT | M | 10 | 1.000±0.2092 | 0.5117-1.458 | ***p=0.0435** |
|  |  | *Nf1^+/-^* |  | 8 | 0.2104±0.052 | 0.08771-0.3331 |  |
|  |  | WT | F | 13 | 1.062±0.1694 | 0.6931-1.432 | p=0.9992 **#p=0.0222** |
|  |  | *Nf1^+/-^* |  | 11 | 1.031±0.1995 | 0.5917-1.470 |  |
|  | *L. reuteri* | WT | M | 10 | 0.983±0.2837 | 0.3411-1.625 | p=0.5126 |
|  |  | *Nf1^+/-^* |  | 8 | 0.2104±0.052 | 0.2583-0.9667 |  |
|  |  | WT | F | 9 | 1.000±0.1620 | 0.6265-1.373 | ***p=0.0466** |
|  |  | *Nf1^+/-^* |  | 12 | 0.4085±0.0630 | 0.2714-0.5456 |  |
|  | *L. rumni* | WT | M | 11 | 1.000±0.2856 | 0.6301-1.644 | p=0.7178 |
|  |  | *Nf1^+/-^* |  | 6 | 0.5778±0.12 | 0.3751-1.015 |  |
|  |  | WT | F | 11 | 1.016±0.1618 | 0.5909-1.493 | p=0.5081 |
|  |  | *Nf1^+/-^* |  | 13 | 1.515±0.3051 | 0.8500-2.179 |  |

Mean values of normalized gene expression, 95% confidence intervals and respective P values obtained by Two-way ANOVA with Tukey's post hoc comparison test for both genotypes according to each sex. CI: Confidence Interval; F: Female; M: Male; N: Sample size; SEM: Standard Error of the Mean *p<0.05 (*Nf1^+/-^* versus WT in the same sex) **p<0.001; ***p<0.0001; ^#^(Male *Nf1^+/-^* versus Female *Nf1^+/-^*)<0.0.5; ^##^p<0.001; ^###^p<0.0001;

**Table5**

Female intestinal *NPY* and receptors expression according to ovulatory cycle.

|  | **Gene/ Specie** | **Genotype** | **Estrous phase** | **N** | **Mean Value**  **± SEM** | **95 % CI of mean** | **P-value** |
| --- | --- | --- | --- | --- | --- | --- | --- |
| **Intestine** | *NPY* | WT | Follicular | 6 | 1.000±0.4212 | -0.08269-2.083 | ******p<0.0001** |
|  |  | *Nf1^+/-^* |  | 5 | 16.66±2.371 | 9.116-24.21 |  |
|  |  | WT | Luteal | 6 | 1.031±0.4715 | -0.2121-2.212 | p=0.9440  **^####^p<0.0001** |
|  |  | *Nf1^+/-^* |  | 3 | 1.909±0.4754 | -0.1359-3.955 |  |
|  | *PYY* | WT | Follicular | 6 | 1.000±0.6040 | -0.5526-2.553 | p=0.6661 |
|  |  | *Nf1^+/-^* |  | 5 | 2.104±0.8309 | -0.2029-4.411 |  |
|  |  | WT | Luteal | 6 | 1.013±0.5193 | -0.3224-2.347 | p=0.1753 |
|  |  | *Nf1^+/-^* |  | 3 | 3.443±1.117 | -1.363-8.249 |  |
|  | *Y2* | WT  *Nf1^+/-^* | Follicular | 6  5 | 1.000±0.7240  6.166±1.924 | -0.8612-2.861  0.8236-11.51 | ***p=0.0433** |
|  |  | WT  *Nf1^+/-^* | Luteal | 6  3 | 1.018±0.4544  4.948±2.556 | -0.1680-2.168  -6.051-15.95 | p=0.2586 |
|  | *Y4* | WT  *Nf1^+/-^* | Follicular | 6  5 | 1.000±0.9560  2.579±1.862 | -1.458-3.457  -2.591-7.749 | p=0.7962 |
|  |  | WT  *Nf1^+/-^* | Luteal | 6  3 | 0.3343±0.2326  4.986±2.272 | -0.2635-0.9321  -4.789-14.76 | p=0.1363 |
| **Microbiome** | Lactobacillus | WT | Follicular | 6 | 1.000±0.6879 | -0.7682-2.768 | p=0.7564 |
|  |  | *Nf1^+/-^* |  | 5 | 0.1924±0.04381 | 0.07076-0.3140 |  |
|  |  | WT | Luteal | 6 | 0.8539±0.7007 | -0.973-2.655 | p=0.8433 |
|  |  | *Nf1^+/-^* |  | 3 | 0.07202±0.0237 | -0.02975-0.1738 |  |
|  | *L. reuteri* | WT | Follicular | 6 | 1.000±0.8151 | -1.263-3.263 | p=0.7548 |
|  |  | *Nf1^+/-^* |  | 5 | 0.1761±0.05063 | 0.03556-0.3167 |  |
|  |  | WT | Luteal | 6 | 1.007±0.6429 | -0.6454-2.66 | p=0.7891 |
|  |  | *Nf1^+/-^* |  | 3 | 0.1450±.01465 | 0.08197-0.2080 |  |
|  | *L. rumni* | WT | Follicular | 6 | 1.000±0.2856 | 0.2657-1.734 | p=0.6290 |
|  |  | *Nf1^+/-^* |  | 5 | 0.5778±0.2956 | -0.2429-1.398 |  |
|  |  | WT | Luteal | 4 | 0.4802±0.1618 | -0.03484-0.9952 | p=0.9247 |
|  |  | *Nf1^+/-^* |  | 3 | 0.2090±0.1388 | -0.3881-0.8061 |  |

Mean values of normalized gene expression, 95% confidence intervals and respective P values obtained by Two-way ANOVA with Tukey's post hoc comparison test for both genotypes according to each estrous cycle. CI: Confidence Interval; N: Sample size; SEM: Standard Error of the Mean
* (*Nf1^+/-^* versus WT in the same estrous cycle) p<0.05; **p<0.001; ***p<0.0001; ^#^(Luteal phase *Nf1^+/-^* versus Follicular *Nf1^+/-^*)<0.0.5; ^##^p<0.001; ^###^p<0.0001;

**
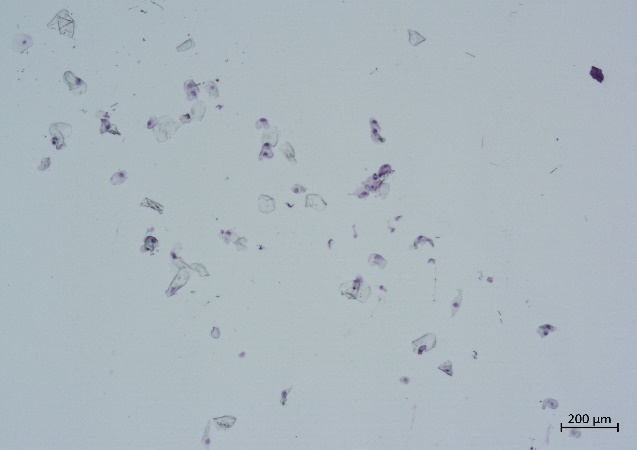

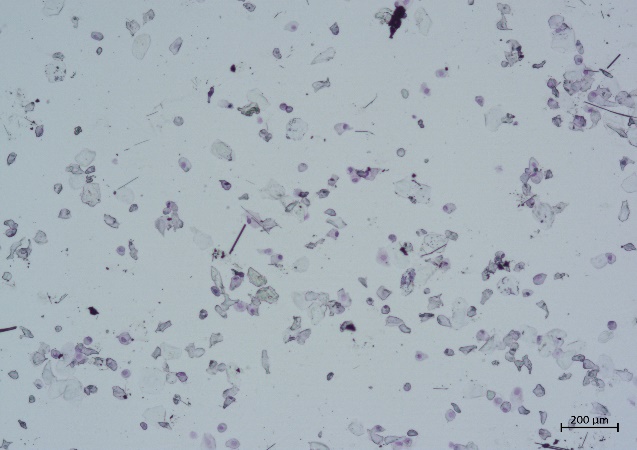
Additional Figure 1**

**A.**

**B.**

**Intestinal** ***NPY* and *Y2* expression are dependent of female menstrual cycle.**

Representative images for follicular (**A**) and luteal (**B**) stage of the estrous cycle at a ×400 magnification.

**Additional Figure 2**

**
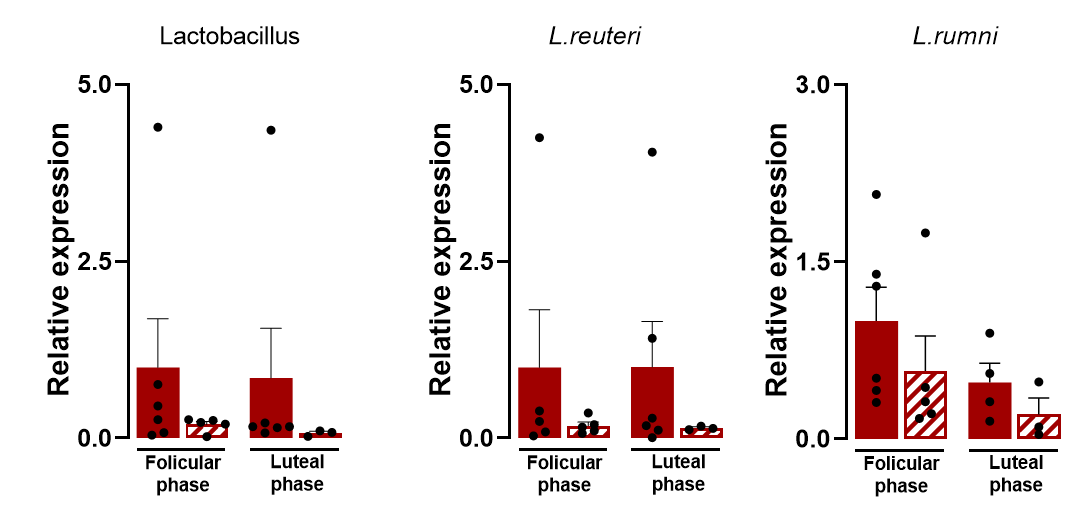
**

No changes were observed in gut microbiota abundance along estrous cycle. Two-way ANOVA with Tukey's post hoc correction analysis. Genotype effect: F_Lactobacillus_ [1, 16] = 1.610, p=0.2226; estrous cycle effect: F_Lactobacillus_ [1, 16] = 0.04526, p=0.8342; interaction: F_Lactobacillus_[1, 16] = 0.0004217 p=0.9839;Genotype effect: F*_L..reuteri_* [1, 15] = 1.841, p=0.1949; estrous cycle effect: F*_L.reuteri_* [1, 15] = 0.0003746, p=0.9848; interaction: F*_L.reuteri_*[1, 15] = 0.0009459 p=0.9759; Genotype effect: F*_L.rumni_* [1, 15] =2.042, p=0.1722; estrous cycle effect: F*_L.rumni_*[1, 16] = 0.008072, p=0.9295; interaction: F*_L.rumni_*[1, 16] = 0.001802 p=0.9667.Data are expressed as mean ± SEM, n =3-6 per group. Statistical significance was found by two-way ANOVA followed by Tukey's post hoc multiple comparisons test. *p < 0.05 and **p < 0.01 (vs WT) and **^#^**p<0.05, **^##^**p<0.01, **^###^**p<0.001 (vs opposite estrous phase).

**Additional Figure 3**

**
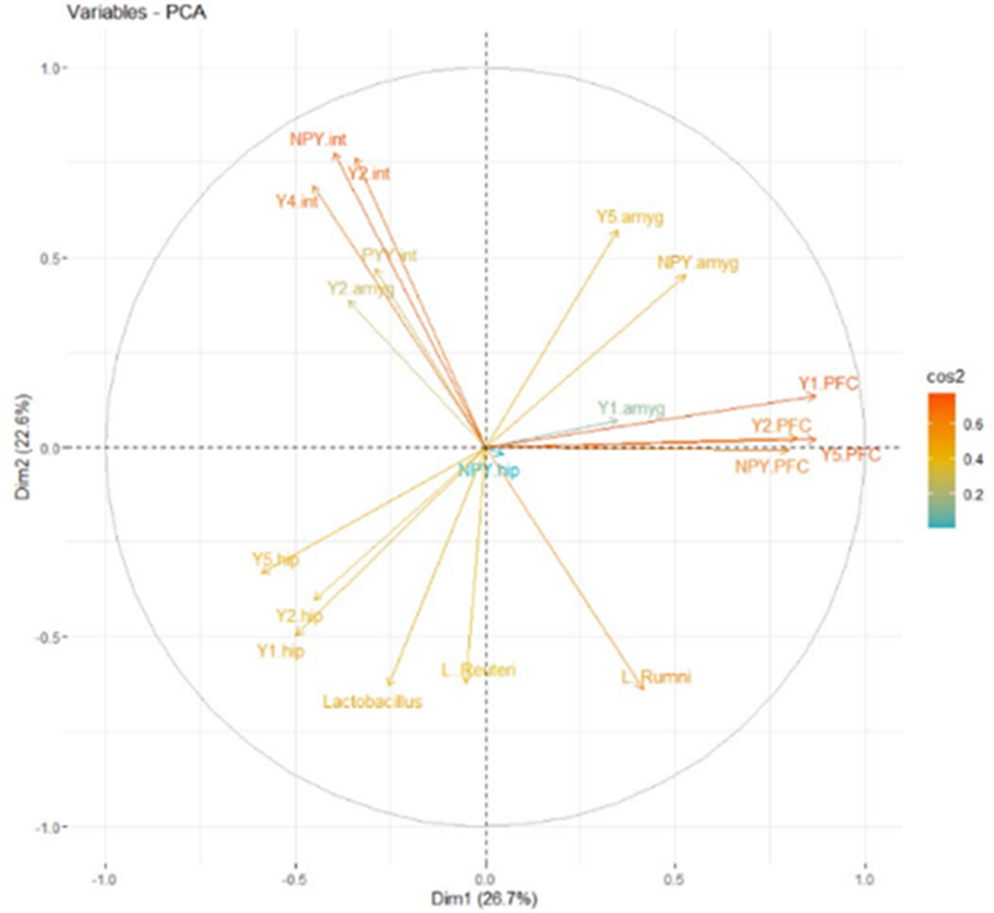
**

The loadings of variables and their relative importance for PCA
